# Report of Henry T. Child, M. D., Vaccine Physician for the Northern District of the Northern Liberties of Philadelphia

**Published:** 1847-02

**Authors:** Henry T. Child

**Affiliations:** No. 132 Green street, N. L.


*Report of* Henry T. Child, M. D., *Vaccine Physician for the Northern District of the Northern Liberties of Philadelphia.*

To the Board of Commissioners of the Northern Liberties.

Gentlemen:—During the quarter ending on the first inst., I vaccinated eighty-one persons, twenty-nine of whom were males, and fifty two were females, which, together with four hundred and forty cases previously reported, makes a total of five hundred and twenty-one cases for the year 1846.

During the last three months I have found that a large number of persons were insusceptible to the vaccine disease, when vaccinated under the most favourable circumstances, probably owing to the fact that we have no epidemic small pox influence in our midst.

In a former report, I remarked that previous to the existence of the epidemic, which was then prevailing, the failures among the first vaccinations were about ten per cent, but at that time they were not more than two per cent. After the disappearance of the epidemic the proportion of failures began to increase, and at present they are over thirty per cent, although the insertion of the virus, (known by its effect on others to be good,) has been repeated several times. I believe it is a fact that in those countries where small pox has never prevailed, vaccination can not be successfully performed, and I am inclined to the opinion that when there is no epidemic small pox existing, many persons will not receive the genuine vaccine disease; hence they will not be properly protected, and when exposed to the influence of small-pox, may be infected with varioloidor the disease in a modified form, and therefore I repeat my conviction as expressed in a former report, "that re-vaccination is necessary, during the existence of epidemic small pox, as a test of the protection of the system." All of which is respectfully submitted.

HENRY T. CHILD.

No. 132 Green street, N. L., 1st mo. 4th, 1847.

